# Ethnic disparities in STEMI outcomes among older adults: a comparative study of bedouins and jews

**DOI:** 10.1186/s12939-025-02427-0

**Published:** 2025-03-10

**Authors:** Sagi Shashar, Vladimir Zeldetz, Aryeh Shalev, Orit Barret, Yan Press, David Shamia, Boris Punchik

**Affiliations:** 1 Clinical Research Center, Faculty of Health Sciences, Soroka University Medical Center, Ben-Gurion University of the Negev, P.O. Box 151, Be’er Sheva, 84101 Israel; 2 Department of Emergency Medicine, Soroka University Medical Center, Beer-Sheva, Israel; 3 Department of Cardiology, Soroka University Medical Centre, Beer Sheva, Israel; 4 Department of Geriatrics, Soroka Medical Center, Beer-Sheva, Israel; 5 Unit for Community Geriatrics, Division of Health in the Community, Ben-Gurion University of the Negev, Beer-Sheva, Israel

**Keywords:** ST-Elevation myocardial infarction (STEMI), Older patients, Ethnicity, Bedouins, Jews, Cardiovascular outcomes, Health disparities, Southern Israel, Healthcare access

## Abstract

**Background:**

ST-Elevation Myocardial Infarction (STEMI) is a critical condition, especially in the older population, who are at increased risk due to comorbidities and delayed diagnosis. This study aimed to investigate the impact of ethnicity on the clinical characteristics, treatment timelines, and outcomes of older patients with STEMI in southern Israel, comparing Jewish and Bedouin populations.

**Methods:**

We conducted a retrospective cohort study at Soroka University Medical Center from 2016 to 2022, including older patients (≥ 65 years) diagnosed with STEMI. Patients were grouped by ethnicity: Jews and Bedouins. Data on demographics, comorbidities, treatment timelines, and clinical outcomes were collected. Statistical analysis included a comparison analysis and a multivariable logistic regression, adjusting for potential confounders.

**Results:**

575 older patients diagnosed with STEMI were included in the study, of them 469 Jews (81.6%) and 106 Bedouins (18.4%). The mean age of the cohort was 74.35 ± 7.33 years, with no significant difference between Jews (74.56 years 7.53) and Bedouins (73.40 ± 5.99 years, *p* = 0.139). Bedouins had higher rates of diabetes (53.8% vs. 40.7%, *p* = 0.019) and smoking (40.6% vs. 27.9%, *p* = 0.015) and were less likely to arrive by ambulance (39.6% vs. 62.5%, *p* < 0.00). Bedouins also experienced longer median times from pain onset to first medical contact (126.5 min vs. 90.0 min, *p* = 0.006) and total ischemic time (240.0 min vs. 205.0 min, *p* = 0.003). Despite these differences, there were no significant differences in in-hospital mortality (13.2% Bedouins vs. 10.9% Jews, *p* = 0.606), 30-day mortality (14.2% Bedouins vs. 11.5% Jews, *p* = 0.556), or one-year mortality (21.7% Bedouins vs. 20.9% Jews, *p* = 0.959). Multivariable analysis confirmed no significant association between ethnicity and mortality outcomes.

**Conclusions:**

Despite the higher prevalence of comorbidities among Bedouin patients, less likely to arrive by ambulance, and experienced longer delays in receiving care, their mortality outcomes were comparable to Jewish patients. These findings highlight the effectiveness of the acute care system in southern Israel. However, further research is needed to explore potential differences in other outcomes, such as quality of life and functional recovery, to better address healthcare disparities in this population.

## Introduction

ST-Elevation Myocardial Infarction (STEMI) remains a critical condition, particularly in the older populations, where the combination of advanced age and comorbidities contributes to increased morbidity and mortality [1]. The importance of investigating STEMI in older patients lies in the unique challenges this population faces, including atypical symptom presentation, delayed diagnosis, and the complexity of managing multiple chronic conditions alongside acute cardiac events [2]. These factors can significantly influence clinical outcomes, making timely and effective treatment crucial [1, 2].

The diverse ethnic landscape presents an additional layer of complexity in understanding and managing STEMI in older patients [3]. The population discussed in this study includes Jews and Arab-Bedouins, a minority group within Israel, referred to hereafter as ‘Bedouins’. These two prominent ethnic groups exhibit distinct cultural, socio-economic, and healthcare access patterns [4] that may influence the presentation, management, and outcomes of STEMI. Bedouins face higher rates of cardiovascular risk factors, such as diabetes [5] and hypertension [6], contributing to poorer health outcomes and a lower life expectancy—76 years for Bedouin males compared to 81 years for Jewish males [7]. While Jews, particularly in urban areas, generally benefit from higher socio-economic status, better healthcare access, and greater health literacy, the Bedouin population, despite no longer being traditionally semi-nomadic, continues to encounter significant barriers to healthcare. These challenges are particularly pronounced in unrecognized villages and towns, where the lack of essential infrastructure limits access to medical services, despite ongoing socio-economic and cultural transitions [8]. Geographic isolation, lower socio-economic status, and cultural practices that may prioritize traditional medicine over modern healthcare further exacerbate these disparities [8].

Therefore, investigating STEMI in older patients within these two distinct ethnic groups is crucial for understanding the broader implications of healthcare disparities and for developing strategies to improve outcomes across diverse populations. By identifying the unique challenges and needs of each group, healthcare providers can better tailor their approaches, ensuring that all patients receive the best possible care, regardless of their ethnic background. This research has the potential to contribute significantly to the field of cardiovascular health, particularly in multicultural and diverse societies.

## Methods

### Study design

This was a retrospective cohort study conducted at Soroka University Medical Center (SUMC) in Beer Sheva, Israel, spanning from 2016 to 2022. SUMC, a 1200-bed tertiary care hospital, serves as the primary healthcare facility for most of the northern Negev region’s population. The study was approved by the institutional ‘Helsinki’ review board (approval number: 0298-23-SOR). Given the retrospective nature of the study, informed consent was waived. All data were retrieved from the institutional database, with strict measures taken to anonymize and encode patient information to ensure confidentiality.

### Study population

The study focused on older patients (aged 65 years and older) who were diagnosed with STEMI and subsequently admitted to the intensive cardiac care unit for urgent catheterization. Patients were grouped according to their ethnicity, classified as Bedouins or Jews. This classification was primarily based on both the patient’s given name and family name, distinguishing between Arabic and Hebrew naming conventions. In cases where ambiguity arose, additional factors were considered, including the patient’s place of residence and the location of their primary healthcare clinic, as most Bedouins reside in cities and villages predominantly inhabited by Bedouins [9]. The study included patients who presented within 12 h of symptom onset, classified as acute coronary syndrome STEMI, as well as those who arrived between 12 and 48 h, classified as evolved STEMI, or after 48 h, classified as recent STEMI. These patients required urgent catheterization due to specific clinical indications, such as ongoing chest pain with ST elevation on ECG, hemodynamic instability, or critical arrhythmias, including ventricular tachycardia, ventricular fibrillation, or complete atrioventricular block [10]. Patients younger than 65 years, those transferred from other hospitals, those evacuated by helicopter, and non-residents of Israel were excluded from the study.

### Data collection

Data were manually extracted and analyzed from computerized records, primarily utilizing the “CHAMELEON” and “CARDIOLOGY DOS” systems, by one of the study’s researcher, a senior physician in the emergency department of SUMC. The information gathered included demographic details including age, ethnicity, and gender, as well as data on pre-existing ischemic heart disease (IHD) and common risk factors like hypertension, dyslipidemia, diabetes mellitus, and smoking status. The Charlson Weighted Comorbidity Index (CWCI) was calculated for each patient. Key timing metrics recorded included the time from symptom onset to first medical contact, hospital arrival, start of catheterization, artery opening, and the total ischemic time from symptom onset to artery opening. Arrival characteristics included the mode of transportation—self arrival, ambulance, or referral from private after hours emergency medical centers such as “Terem” or “Briuta”—as well as whether the patient was transported directly to the catheterization room or first to the hospitals’ ED. Clinical characteristics examined included the presence of cardiac arrhythmias (ventricular tachycardia/ventricular fibrillation, complete atrioventricular block) and cardiogenic shock prior to catheterization. Left ventricular systolic function was assessed via echocardiography within 48 h of admission, and categorized into normal/preserved/mild dysfunction, moderate dysfunction, or severe dysfunction.

### Outcomes

Clinical outcomes included in-hospital mortality, 30-day mortality, and 1-year post-discharge mortality for those who survived and were discharged.

### Statistical analysis

Categorical variables were described as frequencies and percentages, while continuous variables were presented as means with standard deviations (SD) for normally distributed data, or as medians with minimum and maximum values for non-normally distributed data. The study population was divided into two ethnic groups: Bedouins and Jews. Categorical variables were compared using the Chi-squared test, while parametric continuous variables were analyzed with the unpaired Student’s t-test, and non-parametric continuous variables with the Mann-Whitney U test. Univariable and multivariable logistic regression models were employed to explore associations between ethnicity and clinical outcomes. Selection of variables for the multivariable models was guided by previous research, clinical relevance, and findings from the univariable analysis. The regression results were expressed as adjusted odds ratios (AdjORs) with 95% confidence intervals (95% CIs) and associated p-values. Statistical significance was determined at a p-value of less than 0.05, with all analyses performed using RStudio, version 2024.04.2.

## Results

This study included 575 older patients with STEMI, comprising 469 Jews (81.6%) and 106 Bedouins (18.4%). The mean age was 74.35 years (SD = 7.33), with no significant difference between Jews (74.56 ± 7.58 years) and Bedouins (73.40 ± 5.99 years, *p* = 0.139). The proportion of female patients was similar between Jews (149, 31.8%) and Bedouins (33, 31.1%, *p* = 0.991).

Table 1 depicts the descriptive statistics of the entire populations along with the comparisons between groups. Bedouins had a higher prevalence of diabetes mellitus (57, 53.8%) compared to Jews (191, 40.7%, *p* = 0.019). Similarly, smoking was more prevalent among Bedouins (43, 40.6%) than Jews (131, 27.9%, *p* = 0.015). Dyslipidemia was more common in Bedouins (85, 80.2%) than Jews (333, 71.0%), though this difference was not statistically significant (*p* = 0.072). The Charlson Comorbidity Weighted Index (CCWI) had a median of 4.0 for both groups, with a slightly narrower range in Bedouins (min-max: 2.00–7.00) compared to Jews (min-max: 1.00–10.00, *p* = 0.051).

Our analysis revealed that more Jewish patients arrived by ambulance, with 293 out of 469 (62.5%) using this method, compared to 42 out of 106 Bedouin patients (39.6%, *p* < 0.001). Conversely, Bedouins were more likely to arrive on their own or be referred by an emergency medical center, with 21 (19.8%) arriving independently and 43 (40.6%) referred, compared to 86 (18.3%) and 90 (19.2%) of Jewish patients, respectively. Additionally, 72 Bedouin patients (67.9%) were admitted through the ED, versus 246 Jewish patients (52.5%, *p* = 0.005).

Regarding treatment timelines, Bedouins experienced longer delays across all measured intervals. The median time from pain onset to first medical contact was longer in Bedouins (126.5 min, min-max: 12.00–3200.00) than in Jews (90.0 min, min-max: 5.00–3570.00, *p* = 0.006). The median pain-to-door time was 158.5 min (min-max: 28.00–3312.00) for Bedouins and 128.0 min (min-max: 15.00–3637.00) for Jews (*p* = 0.01). The door-to-device time was slightly longer for Bedouins (55.0 min, min-max: 11.00–157.00) compared to Jews (50.0 min, min-max: 5.00–241.00, *p* = 0.035). The median door-to-balloon time did not differ significantly between the groups (Bedouins: 74.5 min, min-max: 20.00–178.00; Jews: 66.0 min, min-max: 15.00–270.00, *p* = 0.155). Overall, total ischemic time was longer in Bedouins (240.0 min, min-max: 80.00–3482.00) than in Jews (205.0 min, min-max: 80.00–3706.00, *p* = 0.003).

Analysis of complications showed no significant differences in left ventricular function (*p* = 0.116) and life-threatening arrhythmias were similarly distributed between Jews (27 with VT/VF, 5.8%; 24 with AV block, 5.1%) and Bedouins (4 with VT/VF, 3.8%; 7 with AV block, 6.6%, *p* = 0.61). Cardiogenic shock prior to PCI occurred in 36 Jews (7.7%) and 10 Bedouins (9.4%, *p* = 0.686).

Outcomes showed no significant differences between Jews and Bedouins in in-hospital mortality (51, 10.9% vs. 14, 13.2%, *p* = 0.606), 30-day mortality (54, 11.5% vs. 15, 14.2%, *p* = 0.556), or one-year mortality (98, 20.9% vs. 23, 21.7%, *p* = 0.959). The median length of stay in the intensive cardiac care unit (ICCU) was 4 days for both groups (Jews: min-max: 1.00–22.00; Bedouins: min-max: 1.00–13.00, *p* = 0.21).

Multivariable analyses, adjusted for age, gender, CCWI, total ischemic time, left ventricular function severity, and the presence of life-threatening arrhythmias and cardiogenic shock, revealed no significant differences in outcomes by ethnicity. The odds ratios for in-hospital mortality (AdjOR = 1.01, 95% CI: 0.44–2.20, *p* = 0.975), 30-day mortality (AdjOR = 1.05, 95% CI: 0.47–2.20, *p* = 0.910), and one-year mortality (AdjOR = 0.84, 95% CI: 0.44–1.55, *p* = 0.585) did not indicate increased risk associated with ethnicity (Table 2).

## Discussion

This study aimed to explore the demographic and clinical characteristics, treatment timelines, and outcomes of older patients diagnosed with STEMI in southern Israel, focusing on comparisons between Jewish and Bedouin populations. Our findings revealed that Bedouin patients had higher rates of diabetes mellitus and smoking compared to Jewish patients and they arrived at the hospital later, with significantly longer delays in both pain-to-first medical contact and pain-to-door times. Despite these differences, there were no statistically significant differences in in-hospital mortality, 30-day mortality, or one-year mortality between the two ethnic groups.

### STEMI in the older population

STEMI in older patients presents unique challenges due to the increased prevalence of comorbidities, atypical presentations, and higher risk of complications Older adults generally experience worse outcomes compared to younger patients, including higher mortality rates [1]. Delays in seeking care, often caused by atypical symptoms or difficulty accessing healthcare, further complicate treatment [1]. This study contributes to the existing body of knowledge by examining these challenges in a regional context, focusing on older patients from Jewish and Bedouin ethnic backgrounds.

### Ethnic disparities in STEMI management in older adults

Ethnic disparities in cardiovascular outcomes have been well-documented globally [3, 11]. For example, research from the United States indicates that African American and Hispanic patients with STEMI often face worse outcomes than White patients, driven by delayed access to care, lower socioeconomic status, and higher prevalence of risk factors such as hypertension and diabetes [12]. Similar patterns are found in the United Kingdom, where South Asian patients with STEMI are reported to have higher mortality rates than White patients due to comorbidities and delayed presentations [13]. Previous research in Israel has highlighted differences in cardiovascular risk factors and healthcare utilization between Jewish and Arab populations [14]. However, there has been limited attention given to older patients, particularly in the Bedouin community. Our study helps to fill this gap by demonstrating that, despite challenges and delays faced by Bedouin patients, their mortality outcomes were comparable to those of Jewish patients.

### Impact of standardized acute care in reducing disparities

Although evidence on reducing ethnic disparities through standardized care is still emerging [15], a study in the United States demonstrated that implementing comprehensive STEMI care protocols significantly reduced disparities in outcomes between Black and White patients by improving key process metrics, such as timely reperfusion therapy [16]. Our findings align with this, as there were no significant differences in short-term mortality between Bedouin and Jewish patients, suggesting that standardized protocols, including adherence to international guidelines for MI care, mitigated some of the disparities observed at presentation. By ensuring uniform care once patients entered the hospital, these protocols helped to neutralize disparities linked to delayed presentation or higher baseline risk factors.

Despite improvements in acute care, many studies continue to emphasize the persistence of disparities in long-term cardiovascular outcomes due to socioeconomic and healthcare access barriers [15, 17, 18]. Interventions such as culturally tailored health education, expanded preventive services, and the integration of telemedicine and mobile health clinics are recommended to reach underserved populations, especially those in rural or socioeconomically disadvantaged areas [18]. Community-based care coordination and patient navigation services are also critical in improving adherence to treatment plans and ensuring better follow-up care, which can help reduce rehospitalization rates and improve overall outcomes [18]. However, focusing solely on mortality as an outcome may overlook other key aspects of patient health, including quality of life [19], functional recovery [20], and rehospitalization rates [21]. Addressing these broader health outcomes is equally important in achieving true equity in cardiovascular care.

### Interventions addressing disparities in STEMI between bedouins and jews

The differences in hospital arrival methods between Jewish and Bedouin patients highlight disparities in healthcare access. Jewish patients were more likely to arrive by ambulance, while Bedouin patients more frequently arrived independently or through referrals, often leading to ED admission rather than direct ICCU entry. These disparities reflect broader access barriers, including limited availability of emergency services, transportation challenges, and a lack of infrastructure in remote and unrecognized Bedouin villages [4]. Cultural factors, such as a reliance on traditional medicine and distrust of institutional healthcare, also contribute to delayed medical intervention [8]. Bedouin patients additionally exhibited higher rates of chronic conditions like smoking and diabetes, which exacerbated their overall health disparities [5]. Addressing these chronic conditions is critical in improving long-term health outcomes and reducing the severity of acute cardiovascular events such as STEMI.

To address these challenges, community outreach programs focusing on early symptom recognition and preventive care are essential [22]. Expanding access to emergency services through improved transportation infrastructure in Bedouin communities [23] and strengthening partnerships with local healthcare providers for direct referrals to specialized care [24] are critical steps. Tailoring these interventions to the unique socio-cultural context of the Bedouin population can help reduce healthcare disparities and improve outcomes in STEMI management.

### Limitations

This study has several limitations that should be considered when interpreting the results. First, the study is retrospective in nature, relying on data extracted from medical records, which may be subject to information bias or incomplete documentation. Second, the sample size, particularly of the Bedouin cohort, was relatively small, which may have limited the statistical power to detect differences in outcomes between the two groups. This small sample size may also contribute to the lack of significant findings despite observed trends. Third, our study focused primarily on mortality outcomes, and other important aspects of patient recovery, such as long-term quality of life, functional status, and rehospitalization rates, were not assessed. These outcomes may reveal disparities that were not captured in our analysis. Finally, the generalizability of our findings may be limited to the specific population and healthcare setting of southern Israel, and caution should be taken when applying these results to other regions or populations with different demographic and healthcare characteristics.

## Conclusion

Our study highlights that while Bedouin older patients with STEMI have a higher prevalence of comorbidities, less likely to arrive by ambulance, and experience longer delays in receiving care, their mortality outcomes are comparable to those of Jewish patients. This finding underscores the effectiveness of the acute care system in southern Israel, yet it also raises questions about potential unmeasured differences in other important outcomes. Future research should explore these areas, with a focus on improving the overall health and healthcare experiences of ethnic minority groups, particularly the older patients, in order to achieve better health equity in the management of STEMI.

**Table 1:** Comparison of demographic, clinical characteristics, timelines and outcomes between Jews and bedouins

	Jews (*n* = 469)	Bedouins (*n* = 106)	Total (*n* = 575)	*p*-value
**Demographics**				
Age, years (mean ± SD)	74.56 (7.58)	73.40 (5.99)	74.35 (7.33)	0.139
Female sex (n, %)	149 (31.8)	33 (31.1)	182 (31.7)	0.991
**Comorbidities**				
CIHD (n, %)	130 (27.7)	38 (35.8)	168 (29.2)	0.123
Diabetes mellitus (n, %)	191 (40.7)	57 (53.8)	248 (43.1)	**0.019**
Hypertension (n, %)	328 (69.9)	78 (73.6)	406 (70.6)	0.531
Dyslipidemia (n, %)	333 (71.0)	85 (80.2)	418 (72.7)	0.072
Smoking (n, %)	131 (27.9)	43 (40.6)	174 (30.3)	**0.015**
CCWI (median [min-max])	4.00 [1.00, 10.00]	4.00 [2.00, 7.00]	4.00 [1.00, 10.00]	**0.051**
**Arrival characteristics**				
Arrival by, n (%)				**< 0.001**
Self	86 (18.3)	21 (19.8)	107 (18.6)	
Ambulance ordered by patient	293 (62.5)	42 (39.6)	335 (58.3)	
Referral by an emergency medical center	90 (19.2)	43 (40.6)	133 (23.1)	
Arrival to ED vs. directly to ICCU, n (%)	246 (52.5)	72 (67.9)	318 (55.3)	**0.005**
**Timelines (minutes)**				
Pain to first medical contact (median [min-max])	90.00 [5.00, 3570.00]	126.50 [12.00, 3200.00]	90.00 [5.00, 3570.00]	**0.006**
Pain to door (median [min-max])	128.00 [15.00, 3637.00]	158.50 [28.00, 3312.00]	135.00 [15.00, 3637.00]	**0.01**
Door to device (median [min-max])	50.00 [5.00, 241.00]	55.00 [11.00, 157.00]	51.00 [5.00, 241.00]	**0.035**
Door to balloon (median [min-max])	66.00 [15.00, 270.00]	74.50 [20.00, 178.00]	67.00 [15.00, 270.00]	0.155
Total ischemic time (median [min-max])	205.00 [80.00, 3706.00]	240.00 [80.00, 3482.00]	210.00 [80.00, 3706.00]	**0.003**
**Complications**				
Left ventricular function (n, %)				0.116
Normal / preserved	71 (15.1)	8 (7.5)	79 (13.7)	
Mild dysfunction	130 (27.7)	26 (24.5)	156 (27.1)	
Moderate dysfunction	144 (30.7)	41 (38.7)	185 (32.2)	
Severe dysfunction	124 (26.4)	31 (29.2)	155 (27.0)	
Life-threatening arrhythmia (n, %)				0.61
No arrythmia	418 (89.1)	95 (89.6)	513 (89.2)	
VT/VF	27 (5.8)	4 (3.8)	31 (5.4)	
High degree AV block	24 (5.1)	7 (6.6)	31 (5.4)	
Cardiogenic shock (n, %)	36 (7.7)	10 (9.4)	46 (8.0)	0.686
**Outcomes**				
In-hospital mortality (n, %)	51 (10.9)	14 (13.2)	65 (11.3)	0.606
30-days mortality (n, %)	54 (11.5)	15 (14.2)	69 (12.0)	0.556
One-year mortality (n, %)	98 (20.9)	23 (21.7)	121 (21.0)	0.959
LOS ICCU (median [min-max])	4.00 [1.00, 22.00]	4.00 [1.00, 13.00]	4.00 [1.00, 22.00]	0.21

• CIHD: Chronic Ischemic Heart Disease

• CCWI: Charlson Comorbidity Weighted Index

• VT: Ventricular Tachycardia

• VF: Ventricular Fibrillation

• LOS: length of stay

• ICCU: intensive cardiac care unit

**Table 2:** Odds for the investigated outcomes by Ethnicity– Results of multivariable analyses

	In hospital mortality			30 day mortality			One year mortality		
*Predictors*	*AdjOR*	*CI*	*p*	*AdjOR*	*CI*	*p*	*AdjOR*	*CI*	*p*
(Intercept)	0.00	0.00–0.01	**< 0.001**	0.00	0.00–0.00	**< 0.001**	0.00	0.00–0.00	**< 0.001**
Bedouins vs. Jews	1.01	0.44–2.20	0.975	1.05	0.47–2.20	0.910	0.84	0.44–1.55	0.585
Females vs. males	0.92	0.44–1.86	0.812	0.90	0.44–1.78	0.769	1.14	0.67–1.93	0.627
Age	1.02	0.97–1.07	0.365	1.04	0.99–1.09	0.093	1.05	1.01–1.09	**0.010**
CWCI	1.30	0.97–1.71	0.071	1.19	0.90–1.56	0.217	1.62	1.30–2.02	**< 0.001**
P2B	1.00	1.00–1.00	**0.006**	1.00	1.00–1.00	**< 0.001**	1.00	1.00–1.00	0.054
LVF	2.79	1.87–4.39	**< 0.001**	2.93	1.98–4.57	**< 0.001**	2.16	1.66–2.87	**< 0.001**
LOS ICCU	1.02	0.91–1.15	0.721	1.00	0.89–1.12	0.968	0.99	0.89–1.10	0.890
Cardiogenic shock prior PCI	21.18	9.86–47.72	**< 0.001**	16.04	7.53–35.61	**< 0.001**	18.84	8.36–46.68	**< 0.001**

CCWI: Charlson Comorbidity Weighted Index; LOS: length of stay; ICCU: intensive cardiac care unit; PCI: percutaneous

## Data Availability

The research data supporting the results of this manuscript are not publicly available due to restrictions imposed by the institutional ethics committee. However, data may be made available upon reasonable request and subject to specific approval by the ethics committee. Requests for data access can be directed to the corresponding author, and will be reviewed in accordance with the ethical guidelines and institutional policies.
